# Long-Term Health and Economic Impact of a Community-Based, Gene-Guided, Nutrition Program: The Sakado Folate Project in Japan

**DOI:** 10.3390/nu18101630

**Published:** 2026-05-21

**Authors:** Yasuo Kagawa, Kaori Sakamoto, Kumiko Shoji, Chiharu Nishijima, Mami Hiraoka

**Affiliations:** 1 Laboratory of Medical Chemistry, Japan Nutrition University, and Institute of Nutritional Science of Japan Nutrition University, 3-9-21 Chiyoda, Sakado City 350-0288, Saitama, Japan; 2 Laboratory of Clinical Nutrition and Dietetics, Japan Nutrition University, 3-9-21 Chiyoda, Sakado City 350-0288, Saitama, Japan; sakamoto@eiyo.ac.jp; 3 Laboratory of Basic Nutrition, Japan Nutrition University, 3-9-21 Chiyoda, Sakado City 350-0288, Saitama, Japan; shoji.kumiko@eiyo.ac.jp; 4 Department of Preventive Gerontology, Center for Gerontology and Social Science, National Center for Geriatrics and Gerontology, 7-430 Morioka-cho, Obu City 474-8511, Aichi, Japan; c-nishijima@ncgg.go.jp; 5 Department of Nutrition, Faculty of Health Care Science, Chiba Prefectural University of Health Science, 2-10-1 Wakaba, Mihama-ku, Chiba City 261-0014, Chiba, Japan; mami.hiraoka_84@cpuhs.ac.jp

**Keywords:** precision nutrition, folate, DHA, community health intervention, cardiometabolic disease prevention, healthcare expenditure

## Abstract

Background/Objectives: Precision nutrition informed by genetic profiling has been proposed to improve public health outcomes; however, long-term, community-based evidence remains limited. This study evaluated the long-term health and economic impacts of the Sakado Folate Project. Methods: Since 2006, residents participating in the Sakado Folate Project received gene-guided nutritional counseling focused on folate intake and related lifestyle factors. Target genes included methylenetetrahydrofolate reductase (*MTHFR*), angiotensinogen (*AGT*), adrenoreceptor B3 (*ADRB3*), and uncoupling protein 1 (*UCP1*); Δ5-fatty acid desaturase (*FADS1*) was incorporated later. Biochemical markers, genetic polymorphisms, and health indicators were monitored longitudinally. Population-level health outcomes and per-capita medical expenditure data were compared with regional and national statistics. Results: In program participants (n = 888), folate status and biochemical indicators improved: 76.1% achieved the serum folate target (≥9.5 ng/mL) and 55.3% achieved the serum total homocysteine target (≤7 μmol/L). Healthier lifestyle behaviors were observed across 99,565 Sakado residents, with the city recording the highest proportion of individuals actively attempting lifestyle improvement (31%) of all districts in the region. Disease prevalence was lower in Sakado City than in Saitama Prefecture overall, at standardized prevalence ratios of 52% for stroke and 86% for cerebral infarction. Per-capita medical expenditure was also lower in Sakado City (¥337,800) than the national average (¥392,044) in 2021. Conclusions: Long-term implementation of a community-based, gene-guided nutritional intervention may improve population health outcomes and reduce healthcare expenditures. Integrating nutrigenomics into public health strategies alongside community education and food environment improvements may contribute to sustainable healthcare systems in aging societies.

## 1. Introduction

The Dietary Reference Intakes for Japanese [1], which form the foundation of national nutritional guidance, provide recommended nutrient intakes stratified by sex, age, and physical activity level. However, these population-based recommendations do not account for interindividual genetic variability. Recent advances in precision nutrition have underscored the importance of tailoring dietary guidance to genetic polymorphisms that affect nutrient metabolism and susceptibility to chronic disease [2]. Where gene–diet interactions exist, uniform dietary recommendations derived from population averages may produce heterogeneous health outcomes, potentially contributing to the observed differences in prevalences of cardiovascular and metabolic diseases across individuals.

Current precision nutrition studies involve thousands of gene polymorphisms, especially single nucleotide polymorphisms (SNPs). However, to provide easily understandable guidance to the approximately 100,000 residents of Sakado City, it is more practical to select a small number of high-frequency SNPs that have a significant impact on cardiovascular metabolic diseases. For example, among SNPs of the folate metabolic pathway, methylenetetrahydrofolate reductase (*MTHFR*) showed a significant association with serum folate concentration and serum homocysteine [3]. The other target genes were angiotensinogen (*AGT*), adrenoceptor B3 (*ADRB3*), and uncoupling protein 1 (*UCP1*) (Table 1). These SNPs are high-frequency and had a significant impact in gene polymorphism studies in the Asia-Pacific region [4]. Particular emphasis was placed on plant- and seafood-derived nutrients, especially folate and docosahexaenoic acid (DHA) because of their established roles in cardiometabolic disease prevention. Thus, SNP of delta5 fatty acid desaturase (*FADS1*) was also studied.

**Figure 1:**
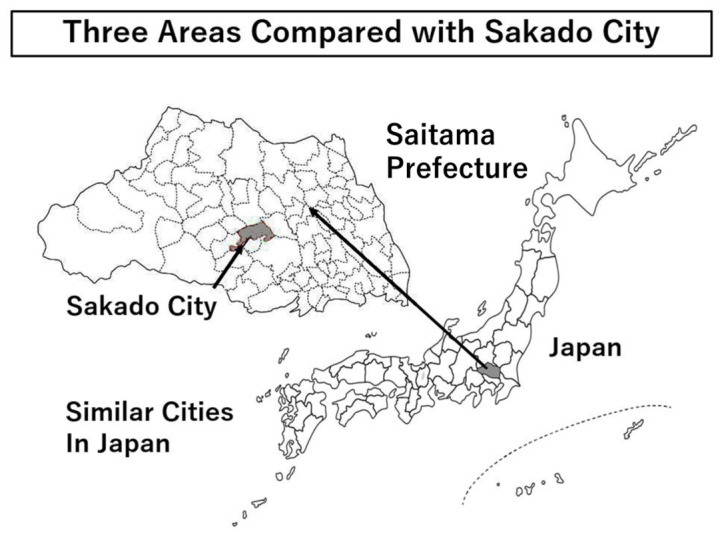
Three areas compared with Sakado City.

Based on this background, a gene-based nutritional guidance program, the Sakado Folate Project, was started in 2006 in Sakado City, Saitama Prefecture, Japan (Figure 1). The person in charge of this project is Yasuo Kagawa, because under Japanese medical regulations, physicians are primarily responsible for the examination of subjects, genetic testing, and disease prevention education. This community-based intervention was designed to promote cardiometabolic health through nutrigenomics-informed dietary guidance.

Folate was prioritized because of its essential roles in DNA synthesis, repair, and methylation [3]. Individuals with the *MTHFR* 677TT genotype characteristically have lower serum folate concentrations and elevated plasma homocysteine levels, a well-established risk factor for cardiovascular disease [3,4]. Previous studies have demonstrated that supplementation with 400 μg/day of folic acid, which is consistent with the WHO recommended intake, effectively improves this metabolic imbalance, whereas the Japanese Dietary Reference Intake of 240 μg/day may be insufficient for this subgroup [4]. A meta-analysis of 21 randomized, controlled trials including 115,559 participants reported that folic acid supplementation significantly reduced the risks of stroke and myocardial infarction [5]. However, reports on improvements in lifestyle behaviors, reductions in medical expenditures, other diseases and genetic polymorphism were lacking in these reports [1,2,3,4,5,6]. A meta-analysis of 95 studies involving 46,175 participants demonstrated that higher dietary folate intake was associated with a decreased risk of incident dementia in older adults [7].

In addition, the Dietary Reference Intakes for Japanese [1] establish recommendations for *n*-3 fatty acid intake under the assumption that DHA can be synthesized endogenously from precursors via Δ5-fatty acid desaturase 1 (*FADS1*). However, individuals carrying the C allele of *FADS1* have markedly reduced enzymatic activity and consequently impaired DHA biosynthesis. In this context, decreasing fish consumption may place individuals with the *FADS1* CC genotype at particular risk of DHA deficiency; accordingly, an intake of 100 g of fish or 1 g of DHA per day was recommended for this subgroup.

Preliminary analyses suggested improvements in lifestyle behaviors and reductions in medical expenditures by participating residents [6,8]. Nevertheless, the long-term, population-level health impact of the intervention has not been comprehensively evaluated with detailed methods and results. Therefore, the present study aimed to assess the 20-year health and economic impact of this community-based, gene-guided nutritional intervention by examining lifestyle indicators, cardiometabolic disease prevalence, and per-capita medical expenditures in Sakado City relative to regional and national reference data [8]. The names of “similar cities” are kept secret, but their population, average age of the citizens and medical care are similar according to reference [8].

## 2. Materials and Methods

### 2.1. Participants, Health Checkups, and Gene-Based Nutritional Targets

**Figure 2:**
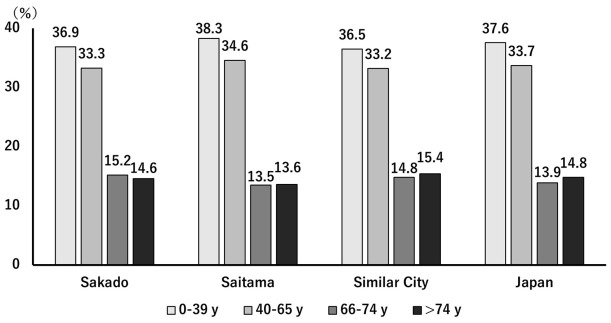
The age distributions of Sakado City, Saitama Prefecture, cities of similar size, and Japan overall. The age distributions are comparable.

The Sakado Folate Project was a community-based, health promotion program targeting all 99,565 residents of Sakado City, Saitama Prefecture, Japan, as of 2024 [6]. The project’s aim was to improve population health through repeated public lectures, social networking services, free medical checkups, and periodic surveys assessing disease prevalence and medical expenditures [6]. Free annual health checkups for all adult and elderly residents included physical examination, measurements of blood pressure and abdominal circumference (for those under 75 years of age), urinalysis, blood tests, and electrocardiography when necessary. The health checkups for the general public conducted by Sakado City were also carried out at specific hospitals. The national health checkup attendance rates for 2015, 2019, and 2024 were 36.0%, 37.5%, and 36.6%, respectively, whereas the rates in Sakado City were slightly higher at 38.8%, 38.9%, and 38.9% [8]. The age distribution of Sakado City was comparable to that of Saitama Prefecture, cities of similar size, and Japan overall (Figure 2) [8], enabling appropriate comparisons of disease incidence, medical costs, and demographic characteristics. For population-level comparisons, data were retrieved from the National Health Insurance Database System [8], the National Health and Nutrition Survey of Japan [9], the Saitama Prefecture Specific Health Checkup Analysis Report [10], and the 3rd Sakado City Health Town Development Plan [11].

In parallel with the population-wide intervention, a representative cohort of 888 participants (144 males, mean age 66.2 ± 9.1 years; 744 females, mean age 63.5 ± 9.1 years) received individualized guidance based on genetic polymorphisms, medical examinations, and dietary assessments. Participants were recruited between 2006 and 2019 by Sakado City from volunteers, introducing a potential selection bias toward individuals who are more health-conscious than the general population. The cohort comprised individuals who completed the intervention program and for whom complete data were available for analysis. Each participant underwent an intervention period of approximately six months and received individualized guidance before and after the intervention. For this program, four candidate SNPs were selected based on mutation frequencies in the Japanese population and evidence linking specific dietary factors to cardiometabolic risk reduction (Table 1). To reduce cardiovascular risk associated with polymorphisms of the *MTHFR* gene, target values were established as follows: serum folate ≥ 9.5 ng/mL and serum homocysteine ≤ 7 μmol/L. Individuals carrying the T allele of *MTHFR* were encouraged to consume 400 μg/day of folate. Those with the T allele of *AGT* were advised to restrict sodium intake to less than 6 g of salt per day. Carriers of the Trp (W) variant of *ADRB3* and the A variant of *UCP1* were advised to reduce body weight through energy restriction combined with increased physical activity. Detailed protocols for gene-specific nutritional counseling have been described previously by our Nutrition Clinic [12].

In response to the marked decrease in fish consumption in Japan, the importance of consuming ≥100 g of fish per day was communicated, particularly for individuals carrying the C allele of Δ5-fatty acid desaturase 1 (*FADS1*), which has been associated with impaired fatty acid metabolism and potential cognitive vulnerability [13], primarily through community-based educational activities. We recommended mackerel, saury, and yellowtail as examples of fish that are rich in DHA and readily available.

All 888 participants received an explanation of the genetic polymorphisms related to folate metabolism and other metabolic risks, and written, informed consent was obtained in accordance with the Declaration of Helsinki. The study protocol was approved by the Ethics Committee of Kagawa Nutrition University (approval numbers No. 222-G, on 26 July 2006 to No. 242-G, on 19 June 2019). Although the project was initiated in 2006 and continued thereafter, the COVID-19 pandemic beginning in 2020 disrupted regular activities. Therefore, analyses of individual-level data for the 888 participants were limited to the period from 2006 to 2019.

### 2.2. Blood Biochemistry

Venous blood samples were collected from the antecubital vein of adult residents of Sakado City in the morning after an overnight fast at a specific laboratory. For the 888 participants, blood biochemical measurements were obtained at baseline and after completion of the six-month intervention program [6]. Blood was drawn into plain tubes and EDTA-containing Venoject tubes. Whole blood was used for genomic DNA extraction as described below. Serum was separated by centrifugation and stored at −80 °C until analysis.

A total of 28 routine biochemical and hematological parameters were analyzed by SRL, Inc. (Tokyo, Japan) according to standardized methods described in the National Health and Nutrition Survey Japan, 2023 Edition [9]. Serum folate and vitamin B_12_ concentrations were measured using a chemiluminescent enzyme immunoassay (Beckman Coulter, Brea, CA, USA). For folate measurement, Access Folate (FOL2) was used from 2006 to 2012, and Access Folate FOLW was used from 2013 onwards. Plasma fatty acids were measured by gas chromatography, as previously described [13]. These analyses were performed by SRL, Inc. From 2006 through 2017, serum total homocysteine concentrations were measured using an enzymatic assay kit (Alfressa Auto Hcy; Alfresa Pharma Inc., Osaka, Japan) according to the manufacturer’s instructions; from 2018 onward, they were measured by SRL using the HPLC method.

### 2.3. Genotyping

Genotyping of all 888 participants was conducted. Genomic DNA was extracted from whole blood using a Magtration System (Precision System Science Co., Ltd., Chiba, Japan) with magnetic bead–based purification [14]. To enable rapid and cost-effective genotyping of a large number of samples, an automated genotyping platform based on a bead-array system within a capillary tube was developed, as previously described [15]. Genotypes of the selected polymorphisms (Table 1) [6,12] and that of *FADS1* [13] were determined using this system and previously described methods. After 2014, genotypes were determined in all samples using TaqMan probes and the 7500 Real-Time PCR system (Applied Biosystems, Foster City, CA, USA).

### 2.4. Public Communications for Health Promotion

Sakado City established the “Sakado City Healthy Community Promotion Ordinance,” consisting of 11 articles that encourage citizens to take proactive steps to improve their own health. Health checkups for Sakado residents included measurements of blood pressure, obesity indices, blood biochemical tests, and urinalysis. When measured values exceeded the normal range, an “H” (high) or “L” (low) indicator was marked on the results sheet. Public health nurses or physicians then provided advice regarding nutrition, exercise, and lifestyle improvement. For the 888 participants who underwent detailed medical examinations, nutritional intake and genetic data (including genes related to folate metabolism and homocysteine regulation) were explained, and individualized nutritional guidance was provided according to the gene polymorphisms shown in Table 1. At the end of the guidance period, the extent of improvement resulting from the intervention was evaluated and reported to the participants. Special lectures on the Sakado Folate Project were delivered at Sakado City Hall three to five times per year. Public awareness activities also included health education programs, printed materials distributed by the city government, and broadcasts via Saitama Television. Health promotion strategies and cost-management approaches were partially modeled on community health center practices in the United States of America [16].

### 2.5. Nutrient Intake and Its Evaluation Using Food Frequency Questionnaires and Diet History Questionnaires

Basic dietary guidance was based on the “Four-Food-Group Point Method” [17], which consists of a combination of foods belonging to four major food groups. These include approximately 240 kcal each from Group I (milk, dairy products, and eggs), Group II (protein sources: meat, fish, and beans), and Group III (sources of minerals, vitamins, and fiber: vegetables and fruits). Total energy intake is then adjusted by varying the intake of Group IV foods (energy sources: grains, oils, and sugars) [17]. The original form of this dietary pattern includes approximately 100 g of fish, 100 g of soybean products, and 400 g of vegetables [17]. This composition is supported by findings from the Dietary Inflammatory Index, which includes approximately 108 g of fish, 99 g of soybeans, and 459 g of vegetables [18]. Therefore, this dietary pattern is suitable for supplying nutrients derived from plant and seafood sources. In addition, simplified guidance using “The Japanese Food Guide Spinning Top,” issued by the Ministry of Health, Labour and Welfare of Japan, was used to explain daily meal composition [19]. In contrast, the average intake of the Japanese population in 2023 was only 58 g of fish, 55 g of soybean products, and 247 g of vegetables per day [9], indicating the need for nutritional assessment and dietary guidance. Food frequency questionnaires (FFQs) are widely used and are considered reasonably valid tools for assessing habitual dietary intake in epidemiological studies [20]. In the present study, dietary intake was assessed using the Self-Administered Diet History Questionnaire (DHQ), which has been validated for use in Japanese populations [21]. DHQ questionnaires were obtained from EBN (Tokyo, Japan), and dietary data were processed using an automated calculation system provided by the developer. This program estimated the intake of approximately 40 nutrients and 150 food items and generated individualized dietary reports for each participant. The primary nutrients evaluated included folate, retinol equivalents (vitamin A), vitamin D, vitamin E, vitamin K, vitamin B_1_, vitamin B_2_, niacin, vitamin B_6_, vitamin B_12_, pantothenic acid, vitamin C, *n*-3 fatty acids, and *n*-6 fatty acids. Food groups were categorized as cereals, potatoes, sugars and sweeteners, pulses, green-yellow vegetables, other vegetables, fruits, fish and shellfish, meat, eggs, milk and dairy products, fats and oils, confectioneries, and seasonings/spices. This general nutritional guidance was modified according to the four gene polymorphisms shown in Table 1 for the 888 participants receiving individualized counseling, based on their nutritional intake assessed by DHQs and their exercise habits.

For the general population of Sakado City, target nutrient intakes listed in Table 1 (e.g., folate 400 μg/day) and DHA intake were broadly recommended.

### 2.6. Statistical Analysis

Statistical analyses were performed using IBM SPSS Statistics version 21 (IBM Corp., Armonk, NY, USA) and JMP version 18 (SAS Institute Inc., Cary, NC, USA). All statistical tests were two-sided, and a *p*-value < 0.05 was considered statistically significant. To compare serum folate and total homocysteine concentrations before and after the intervention by *MTHFR* C677T genotype, a linear mixed-effects model for repeated measures was used. As these variables were not normally distributed, log transformation was applied prior to analysis. Subject was included as a random effect to account for within-individual correlation. Fixed effects included time (before vs. after), genotype, and their interaction, with adjustment for age, sex, and assay method. Estimated marginal means (least-squares means) and their 95% confidence intervals were obtained on the log scale and then back-transformed to the original scale for presentation. The significance of the interaction term was assessed to evaluate whether changes over time differed by genotype. When appropriate, post hoc multiple comparisons were performed using Tukey’s honestly significant difference (HSD) test. Differences in categorical variables were evaluated using the chi-square test. For analyses of *FADS1* gene polymorphisms and related biochemical and dietary variables in a subset of participants, variables that were not normally distributed were analyzed using non-parametric methods. These data are presented as medians. Differences among genotype groups were assessed using the Kruskal–Wallis test, followed by the Steel–Dwass test for multiple comparisons.

## 3. Results

### 3.1. Effects of Health Lectures on Lifestyle Modification

**Figure 3:**
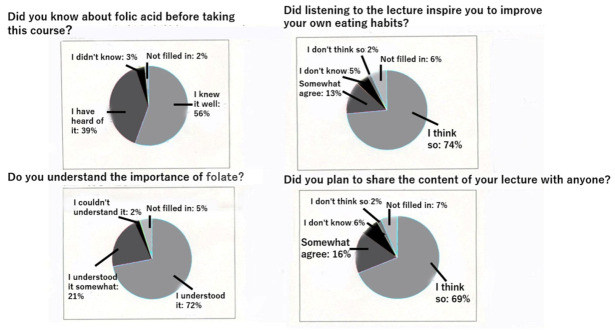
Survey results regarding health education lectures for Sakado citizens (100% response rate, 2025).

**Figure 4:**
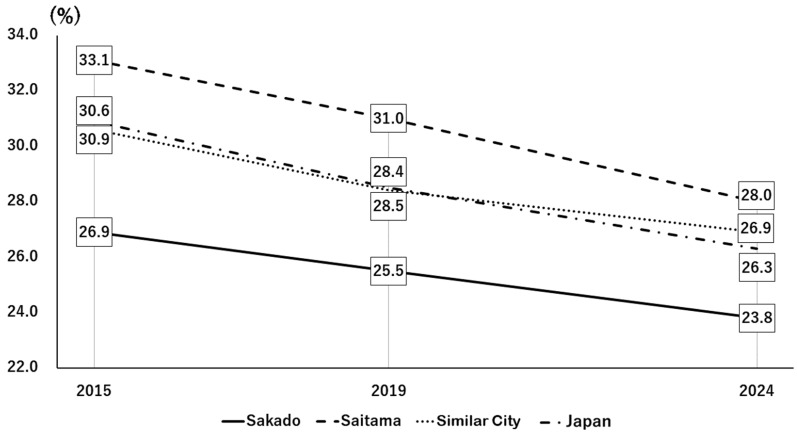
Annual trends in the percentage of residents in four regions who lack the motivation to improve their lifestyle habits.

**Figure 5:**
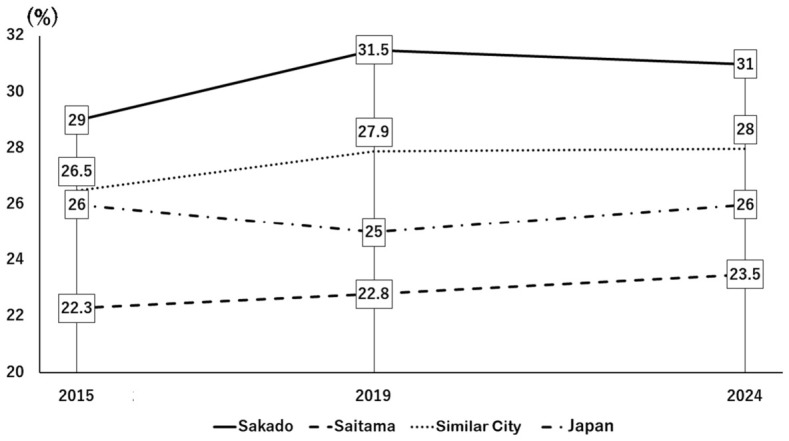
Annual trends in the percentage of residents in four regions who are motivated to improve their lifestyle habits.

The initial stage of the intervention was group instruction through lectures. A survey conducted 19 years after the project was started demonstrated substantial awareness of folic acid among residents of Sakado City. After the lecture, we had all the audience members fill out a questionnaire and then compiled the results. Specifically, 56% of respondents reported familiarity with folate, and 72% responded that they understood its importance (Figure 3, left). In addition, 74% of lecture attendees reported that the lectures motivated them to improve their dietary habits and to share that information with others (Figure 3, right). As a result of understanding the lecture, Figure 4 and Figure 5 show the high level of motivation for health among Sakado citizens. National health guidance data from 2015 to 2024 indicated a gradual decrease in the proportion of individuals who reported no intention to improve their lifestyle habits (Figure 4) [8]. It will be noted that the national data and the data sources for other cities shown in Figure 4 and Figure 5 are from the National Health Insurance Federation and are comparable. Of the comparison groups, Sakado City consistently showed the lowest proportion, followed by Japan overall, comparable municipalities, and Saitama Prefecture (Figure 4) [8]. Conversely, the proportion of individuals actively attempting to improve their lifestyle habits increased modestly over time, with Sakado City showing the highest percentage (Figure 5) [8]. Consistent with these findings, lifestyle behaviors in Sakado City residents were generally healthier than those observed nationwide. The proportions of breakfast skippers and smokers were lower, whereas the proportions of individuals consuming balanced meals and engaging in regular exercise were higher (Table 2) [10,11].

### 3.2. Effects of Genotype Notification on Serum Folate and Homocysteine Levels

**Figure 6:**
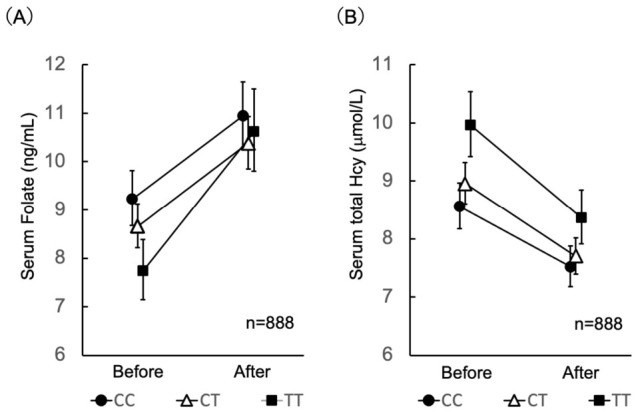
Adjusted changes in serum folate (**A**) and total homocysteine (**B**) concentrations before and after nutritional guidance according to MTHFR C677T genotype. Values are estimated marginal means (least-squares means) derived from a linear mixed-effects model for repeated measures, with subject as a random effect and time (before/after), genotype, and their interaction, as well as age, sex, and assay method as fixed effects. Error bars indicate 95% confidence intervals. A significant interaction between time and genotype was observed for serum folate (*p* = 0.0022), whereas no significant interaction was found for total homocysteine (*p* = 0.1912). The analysis included 888 participants (CC, n = 299; CT, n = 437; TT, n = 152).

Genotype information was provided to the participants together with the nutritional recommendations shown in Table 1. The frequencies of the GG, GA, and AA genotypes of the *AGT* gene in the Japanese population were 7.0%, 30.0%, and 63.0%, respectively [22]. In the present participants, the frequencies of the CC, CT, and TT genotypes of the *MTHFR* polymorphism were 33.7%, 49.2%, and 17.1%, respectively, similar to those reported for the Japanese population (32.8%, 51.6%, and 15.6%, respectively) (Table 1) [23]. The target value for serum folate was set at ≥9.5 ng/mL. Before the intervention, 63.4% of participants met this target, whereas after the intervention 76.1% achieved the target value (*p* < 0.001) [11]. The target value for serum total homocysteine was set at ≤7 μmol/L. Before the intervention, 33.1% of participants met this target, whereas after the intervention, 55.3% achieved the target value (*p* < 0.001) [11]. Changes in serum folate and total homocysteine concentrations before and after nutrition guidance according to MTHFR C677T genotype are shown in Figure 6. Serum folate concentrations increased after the intervention in all genotype groups, with a significant interaction between time and genotype (*p* = 0.0022), indicating that the magnitude of increase differed among genotypes. Serum total homocysteine levels decreased after the intervention across all genotype groups. No significant interaction between time and genotype was observed (*p* = 0.1912), indicating that the magnitude of reduction was similar among genotypes. Although TT genotype showed higher homocysteine levels at baseline, levels decreased after the intervention in all groups. Other risk-related polymorphisms common in the Japanese population, including *ADRB3* [12] and *UCP1* [12], were also disclosed to participants, together with the recommended target values summarized in Table 1. Considering the potential adverse effects associated with reduced intake of long-chain *n*-3 fatty acids [13], increased DHA intake was recommended, particularly for individuals carrying the C allele of the *FADS1* gene. The frequencies of the TT, TC, and CC genotypes of the *FADS1* gene in the Japanese population were 40%, 46%, and 14%, respectively [13].

### 3.3. Effects of Nutrigenomic Guidance on Clinical Data

Comparison of clinical data from the National Health and Nutrition Survey Japan 2023 [9] with those from the Saitama Prefecture Specific Health Checkup Data [10] showed generally similar values (Table 3). However, most clinical indicators, including blood pressure, fasting blood glucose, HbA1c, and LDL-cholesterol levels, were better in Sakado City than in Saitama Prefecture (Table 3) [10]. Since plasma fatty acids, *FADS1* genotypes, folate and homocysteine levels are not analyzed in the National and local Health Checkup, these analyses were conducted in a subset of participants in the Sakado Folate Project (Table 4).

### 3.4. Effects of Nutrigenomic Guidance on Disease Prevalence

The prevalence levels of lifestyle-related diseases, including obesity, hypertension, diabetes mellitus, and dyslipidemia, were significantly lower in Sakado City than in three comparable cities in Saitama Prefecture with younger populations than Sakado City (Table 5) [24]. In 2024, the prevalence levels of hypertension, heart disease, and musculoskeletal disorders were also the lowest in Sakado City compared with Saitama Prefecture, similar cities, and Japan overall [8]. In 17,566 Japanese individuals, the frequencies of the TT, TC, and CC genotypes of the *FADS1* rs174547 polymorphism were 40%, 46%, and 14%, respectively [25]. Similar genotype distributions were observed in 95 subjects in 2014 (Table 4). The prevalence of Alzheimer’s disease in 2024 was also the lowest in Sakado City (15.7%), compared with Saitama Prefecture (16.8%), similar cities (17.7%), and Japan overall (17.4%) [8]. As a result, the rate of nursing care certification in 2024 was 17.0% in Sakado City, compared with 20.1% in Japan overall [8].

### 3.5. Effects of Nutrigenomic Guidance on Medical Costs

**Figure 7:**
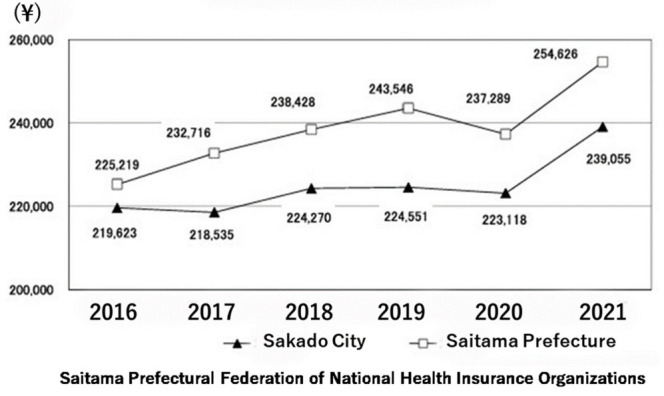
Medical expenses in Sakado City and Saitama Prefecture (per person, per year): 2016–2021.

**Figure 8:**
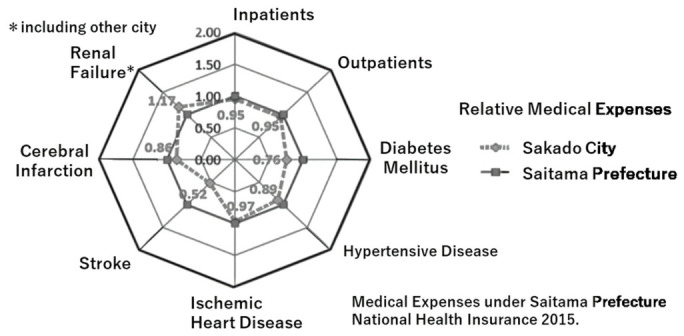
Relative medical expenses in Sakado City (2015) compared with the average annual per-capita medical expenses in Saitama Prefecture for various diseases (set as 1). Hemodialysis for patients with kidney failure is very expensive, so Sakado City subsidizes the costs for patients from another city. That is why a lot of medical expenses are spent on kidney failure.

The medical cost per person in Saitama Prefecture has been the lowest of all prefectures in Japan during the past eight years. Furthermore, medical costs per person were lower in Sakado City than in Saitama Prefecture from 2016 to 2021 (Figure 7) [8]. A consistent longitudinal graph from 2006 to 2025 is impossible to summarize in a single graph because biochemical measurement methods, disease definitions, medical-related laws, etc., have changed many times over this long period. There are many other published figures showing the trends before that period, but in short, for example, in 2011, Sakado City’s cost was ¥192,598, which is slightly cheaper than Saitama Prefecture’s ¥200,104, and it is clear that this difference has widened due to the continuation of the Folate Project. A comparison of the breakdown of medical expenditures between Sakado City and Saitama Prefecture showed that Sakado City had lower expenditure rates for many diseases, including stroke (52%) and diabetes mellitus (76%) (Figure 8) [26]. The only exception was kidney failure; however, this may reflect the transfer of medical expense burdens to Sakado City to avoid the higher costs of private medical care (Figure 8, upper left) [26].

## 4. Discussion

### 4.1. Principal Findings

**Figure 9:**
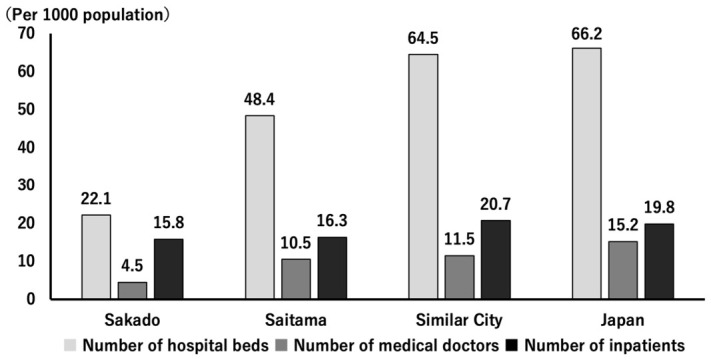
Numbers of hospital beds, doctors, and inpatients per 1000 people in Sakado City, Saitama Prefecture, other cities of similar size, and all of Japan.

The present study demonstrated that a community-based, gene-guided, nutritional program, implemented continuously over a 20-year period through repeated short-term interventions and ongoing public health education, might have improved lifestyle behaviors (Figure 4 and Figure 5, Table 2) and population health indicators (Table 3), as well as decrease disease prevalence (Table 5) and healthcare expenditures (Figure 7 and Figure 8). Intervention of folate status based on personalized nutrition by notifying individuals of their genotype was effective in motivating individuals to change their lifestyle particularly in individuals with the TT genotype [23]. Public health campaigns are being appropriately implemented throughout Japan under the guidance of the Ministry of Health, Labour and Welfare, but Sakado City is the only city that provides health guidance based on SNPs, and it has significantly fewer diseases and lower medical expenses compared to all other areas shown in Figure 1. Guidance based on genetic polymorphisms, such as intake of folic acid-rich rice based on *MTHFR* polymorphism, fish intake based on *FADS1*, salt restriction based on *AGT*, and maintaining a healthy weight based on *ADRB3* and *UCP1*, is significantly different from general public health campaigns in other regions of the country. Over a 20-year period, the “Sakado Folate Project” [6] and the “Shokuiku (Food and Nutrition Education)” program for elementary and junior high school students, which started in 2006 [27], promoted dietary patterns rich in folate and DHA while providing individualized lifestyle guidance based on genetic polymorphisms (Table 1) associated with cardiometabolic risk. Residents of Sakado City exhibited good lifestyle indicators (Figure 4 and Figure 5, Table 2), increased folate intake, improved biochemical markers, and a lower prevalence of several cardiometabolic diseases compared with regional and national averages (Table 3). These improvements were accompanied by substantially lower per-capita medical expenditures (Figure 7 and Figure 8), suggesting that SNPs based nutritional advice might have provided measurable economic benefits at the population level. Notably, Sakado City has relatively low numbers of physicians and hospital beds, representing approximately 29.6% and 30.0% of the national averages, respectively (Figure 9). Therefore, the lower prevalence of disease and reduced medical expenditures observed in Sakado City are unlikely to be explained by greater medical resource availability. Furthermore, the consultation rate in Sakado City (659) is comparable to that of Japan overall, Saitama Prefecture, and similar municipalities, suggesting that the lower disease prevalence is not attributable to underdiagnosis. The age distribution of Sakado City is also comparable to that of Saitama Prefecture, similar cities, and Japan overall (Figure 2). Thus, the lower disease incidence and reduced medical expenditures cannot be explained by demographic differences in population aging. Taken together, these findings suggest that lifestyle improvement through community-based nutritional guidance may have played an important role in improving health outcomes in this population.

### 4.2. Biological Mechanisms

Several biological mechanisms may help explain the observed findings. Folate plays a central role in one-carbon metabolism, DNA methylation, and homocysteine regulation [23,28]. Individuals carrying the *MTHFR* 677TT genotype typically exhibit reduced enzyme activity, leading to elevated homocysteine levels and increased cardiovascular risk [23,28]. Adequate folate intake has been shown to normalize homocysteine metabolism and reduce vascular risk in such individuals [23,28]. Similarly, dietary DHA derived from fish may improve lipid metabolism and inflammatory profiles, particularly in individuals carrying *FADS1* variants that affect fatty acid metabolism. Because the rapid decrease in fish intake began around 2015, and its adverse effects have become increasingly evident [13], nutrigenomic guidance was expanded to include polymorphisms of the *FADS1* gene [25]. Although the serum folate, serum total homocysteine, and intakes of fish, *n*-3 fatty acids, eicosapentaenoic acid (EPA), and DHA were not significantly different among *FADS1* genotypes, linoleic acid was high in the CC genotype, and arachidonic acid was high in the TT genotype (Table 4). One potential molecular mechanism is the homocysteine-lowering effect of DHA through increased expression of cystathionine-γ-lyase [29,30]. Previous randomized trials and meta-analyses have reported that folic acid supplementation reduces the risks of stroke and cardiovascular disease [5], and emerging evidence suggests that higher folate intake may also reduce dementia risk [7]. The present study extends these findings by demonstrating the potential benefits of integrating nutrigenomics-informed dietary guidance into a long-term community health program under real-world conditions.

### 4.3. Public Health Implications

Regarding the accuracy of the study, after rigorously investigating a vast amount of test data, disease frequency, and medical expenses over 19 years, the Japanese Vitamin Society awarded us the Japanese Vitamin Society Award titled “*Nutritional Intervention for Folate-Related Gene Polymorphism-Based Health Promotion Support-Sakado Folate Project*.” In addition, the Governor of Saitama Prefecture, who has a rigorous grasp of the accuracy of Saitama Prefecture’s health surveys and medical expenses, awarded Sakado City the “Excellent Award for Healthy Longevity” in 2024. The folic acid fortification of cereals (140 μg/100 g) in United States of America reduced both diseases and medical cost [31]. The benefit was estimated to prevent 88,172 myocardial infarctions and 1423 neural tube defects per year [31].

Traditional dietary guidelines [1] are designed for the general population and may not fully account for genetic diversity in nutrient metabolism [2,4]. Incorporating genetic information into dietary guidance may therefore enhance the effectiveness of lifestyle interventions and promote greater engagement in health-promoting behaviors [6,31]. In Sakado City, only about 39% of 99,565 residents have undergone health checkups and received guidance on nutrition, exercise, and lifestyle improvement. This uses the percentage of patients who visited the clinic in 2024 as an example, as cited in reference [11]. A major challenge has therefore been how to reach the remaining 61% of residents who do not participate in health checkups. Digital health technologies may help address this issue. Smartphone-based interventions have demonstrated modest but statistically significant effects on weight loss and body mass index reduction over 4–6 months in individuals who are overweight or obese [32]. Lifestyle-related diseases such as diabetes mellitus, hypertension, dyslipidemia, and obesity can be prevented to a considerable extent when individuals are motivated to improve their lifestyle habits (Table 5). In Sakado City, the proportion of residents actively attempting to improve their lifestyle was the highest among the comparison regions (Figure 5), whereas the proportion of individuals with no intention of improving their lifestyle was the lowest (Figure 4). These behavioral differences may have contributed substantially to the lower prevalence of lifestyle-related diseases in Sakado City (Table 5). However, folate intake in Japan decreased from 311 μg/day in 2001 to 282 μg/day in 2012 and further to 270 μg/day in 2023 [9]. In contrast, the average folate intake in the United States of America reached 499 ± 5.4 μg/day in 2017 [33], largely due to mandatory folic acid fortification of cereals [31].

The original form of our dietary guide (fish 100 g, soybean products 100 g, and vegetables 100 g) [17] was shown to be the best combination for the dietary inflammatory index [18], and “Plant and Seafood Nutrients for Potentiating Cardio-Metabolic Disease Prevention”, when modified according to individuals’ genetic polymorphisms (Table 1). The Sakado Folate Project suggests that community-based education, collaboration with local food services, and improved access to nutrient-rich foods (folate-fortified rice, etc.) may facilitate the implementation of nutrigenomics-informed public health strategies.

### 4.4. Strengths and Limitations

This study has several strengths. It represents one of the longest community-based nutrigenomics interventions reported to date, and it integrated genetic testing, dietary assessment, biochemical measurements, and population health indicators. Another strength is the detailed analysis of residents’ motivation to improve their lifestyle behaviors (Figure 5) [24], a key factor in preventing lifestyle-related diseases such as obesity, diabetes mellitus, hypertension, and dyslipidemia (Table 5) [24]. Importantly, the study also evaluated both health outcomes and economic indicators, providing a comprehensive perspective on the potential societal benefits of precision nutrition.

However, several limitations should be considered. First, the study was conducted in a single Japanese city, and the findings may not be fully generalizable to populations with different genetic backgrounds or dietary patterns. For example, dietary patterns in Japan differ substantially from those in the United States of America [1,9,33]. Second, although comparisons were made with regional and national data, causal relationships cannot be established because of the observational nature of the population-level analysis. Third, long-term health outcomes should be expressed in life expectancy and quality-adjusted life years. These important outcomes have been discussed from the standpoint of potential cost-effectiveness of personalized nutrition interventions [34,35]. The socioeconomic status of the 100,000 Sakado citizens is diverse, access to medical care is limited, and lifestyle habits are diverse among the 100,000 citizens. Future studies involving diverse populations and randomized interventional designs will be important to further confirm the effectiveness of nutrigenomics-based, public health interventions.

## 5. Conclusions

This study provides evidence that the long-term implementation of a community-based, gene-guided, nutritional program delivered through repeated short-term interventions and ongoing community-wide health education might have improved population health outcomes and reduce healthcare expenditures. The Sakado Folate Project integrated nutrigenomics-informed dietary guidance with broader community health promotion and might have led to improved folate status, biochemical markers, healthier lifestyle behaviors, and a lower prevalence of cardiometabolic diseases at the population level. The program placed particular emphasis on plant- and seafood-derived nutrients, notably folate and docosahexaenoic acid, which may have contributed to improvements in metabolic and vascular health. In addition, per-capita medical costs were substantially lower than both regional and national averages. However, causal relationships cannot be established because of the observational nature of the population-level analysis. Collectively, the present findings highlight the potential of precision nutrition as a public health strategy for chronic disease prevention and for supporting sustainable healthcare systems in aging societies.

## Figures and Tables

**Table 1:** Risk genes analyzed and used for nutritional advice.

Risk Gene Abbreviation	rs Number	Mutant Hetero %	Homo %	Nutritional Advice
**Angiotensinogen** ***AGT***	rs4762 (G > A)	GA 25%	AA 72%	Restrict salt < 6 g/day
**Methylene tetrahydro folate reductase *MTHFR***	rs180113 (C > T)	CT 51.6%	TT 15.6%	Take folate > 400 μg/day Serum folate > 9.5 ng/mL Serum homocysteine < 7 μmol/L
**Adrenoreceptor B3** ***ADRB3***	rs4994 (W > R)	WR 25.0%	RR 5.0%	Reduce caloric intake 200 kcal/day
**Uncoupling protein 1** ***UCP1***	rs1800592 (A > G)	AG 43.0%	GG 52.0%	Reduce caloric intake 200 kcal/day

**Table 2:** Comparison of the frequencies of four types of lifestyle habits between residents of Sakado City and residents of Japan as a whole.

	Sakado Citizens **		Japanese Overall ***	
	Male	Female	Male	Female
**Breakfast skippers**	10.4%	7.5%	15.5%	11.1%
Balanced meal * > twice per day	74.1%		45.7%	47.1%
**Exercise 30 min > twice a week**	55.0%		28.5%	22.7%
**No smoking**	81.2%		74.4%	93.1%

* A meal with a staple food, main dish, and side dish. ** *p* < 0.01: Sakado and Japan. *** The National Health and Nutrition Survey Japan (2023) [9].

**Table 3:** Comparison of clinical test values from mass health screenings in Japan, Saitama Prefecture, and Sakado City.

Laboratory Test Value	Male			Female		
	Japan	Saitama	Sakado	Japan	Saitama	Sakado
**BMI (** **kg** **/m** **^2^** **)**	23.6 ± 3.6	24.30 ± 3.74	Low	22.4 ± 3.8	22.56 ± 3.97	High
**Sys Pre (mmHg)**	131.6 ± 17.7	129.2 ± 17.0	Low	126.2 ± 19.1	125.5 ± 18.4	Low
**Dia Pre (mmHg)**	77.2 ± 11.4	79.7 ± 11.8	Low	73.6 ± 10.4	74.6 ± 11.5	Low
**FBG (mg/dL)**	104.7 ± 31.5	102.1 ± 23.8	S. Low	98.4 ± 20.4	94.6 ± 16.2	Low
**HbA1c (%)**	5.6 ± 0.6	5.78 ± 0.81	S. Low	5.5 ± 0.4	5.67 ± 0.57	S. Low
**LDL-C (mg/dL)**	109.6 ± 31.9	122.7 ± 31.9	Low	118.1 ± 31.4	124.8 ± 31.2	S. Low
**eGFR**	68.9 ± 15.6	73.49 ± 14.81	No D *	71.3 ± 17.4	73.48 ± 14.44	No D *

Data were obtained from The National Health and Nutrition Survey Japan (2023) [9] and the Saitama Prefecture Specific Health Checkup Data Analysis Report (2022) [10] Abbreviations: BMI: Body Mass Index, Sys Pre: Systolic Blood Pressure, Dia Pre: Diastolic Blood Pressure, FBG: Fasting Blood Glucose, HbA1: hemoglobin A1c, LDL-C: Low-Density Lipoprotein Cholesterol, eGFR: estimated GFR. Comparison of Sakado with Saitama: Low: Lower than Saitama. S. Low: Significantly Lower. High: Higher than Saitama. Unit of eGFR, (X): (mL/min/1.73 m^2^). * No D: No data.

**Table 4:** Serum folate, homocysteine, fish intake, EPA intake, DHA intake, and serum long-chain unsaturated fatty acid concentration according to the *FADS1* rs174547 gene polymorphism. Characteristics of postmenopausal women (n = 95) according to *FADS1* rs174547 genotypes.

			All	TT	TC	CC	P ^1^	P ^2^
**Number**			95	36	50	9		
**Serum Folate**		ng/mL	12.6	12.0	12.9	13.0	0.534	
**Serum total Hcy**		μmol/L	7.5	7.9	7.0	8.2	0.468	
**Dietary intake**								
	Total Energy	kcal	1944	2016	1939	1639	0.956	
	Fish intake	g/1000 kcal	35.7	37.9	36.5	25.4	0.063	
	*n*-3 fatty acids	g/1000 kcal	1.47	1.50	1.41	1.23	0.268	
	EPA	g/1000 kcal	0.13	0.15	0.13	0.12	0.134	
	DHA	g/1000 kcal	0.22	0.25	0.22	0.18	0.185	
**Blood Fatty Acids**								
*n*-6	Linoleic acid	%/total fatty acids	28.79	27.39	29.78	30.14	0.037	
	Arachidonic acid	%/total fatty acids	6.20	6.78	6.12	4.60	<0.0001	TT > TC > CC
*n*-3	α-Linolenic acid	%/total fatty acids	0.86	0.85	0.86	0.98	0.137	
	EPA	%/total fatty acids	2.45	2.18	2.78	2.13	0.105	
	DHA	%/total fatty acids	5.09	4.77	5.26	4.93	0.027	TT < TC

All values are expressed as medians. ^1^ P for difference calculated using Kruskal–Wallis test (*p* < 0.05). ^2^ Multiple comparisons calculated using Steel-Dwass test (*p* < 0.05). Abbreviations: Hcy: homocysteine, TT/TC/CC: genotypes of the MTHFR polymorphism, EPA: eicosatetraenoic acid, DHA: docosahexaenoic acid.

**Table 5:** Comparison of the frequency of obesity, hypertension, diabetes mellitus, and dyslipidemia in Sakado City and three other cities with a younger average age than Sakado City.

Disease	Three Younger Cities	Sakado City
**Obesity**	15.2%	14.2%
**Hypertension**	15.8%	12.3%
**Diabetes mellitus**	5.9%	4.7%
**Dyslipidemia**	27.0%	24.6%

Significance *p* < 0.04 in males. In females, *p* < 0.04 except for obesity [24]. Data were obtained from a nutrition survey conducted by the Saitama Prefecture Department of Health in 2011 and show the prevalence (% of adult male population) of four diseases in the analyzed cities.

## Data Availability

The data presented in this study are included in the article. Further inquiries can be directed to the corresponding author.

## Abbreviations

The following abbreviations are used in this manuscript:

*AGT*	angiotensinogen
*ADRB3*	adrenoreceptor B3
BMI	body mass index
DBP	diastolic blood pressure
DHA	docosahexaenoic acid
*FADS1*	Δ5-fatty acid desaturase 1
FBG	fasting blood glucose
HbA1c	hemoglobin A1c
LDL-C	low-density lipoprotein cholesterol
*MTHFR*	methylene tetrahydro folate reductase
SBP	systolic blood pressure
*UCP1*	uncoupling protein 1

## References

[1] Ministry of Health, Labor and Welfare (2025). Report of the Committee for the Development of Dietary Reference Intakes for Japanese (2025 Edition).

[2] Mullins V.A., Bresette W., Johnstone L., Hallmark B., Chilton F.H. (2020). Genomics in personalized nutrition: Can you “eat for your genes”?. Nutrients.

[3] Joint FAO/WHO Expert Consultation on Human Vitamin and Mineral Requirements (1998). Vitamin and Mineral Requirements in Human Nutrition: Report of a Joint FAO/WHO Expert Consultation.

[4] Hiraoka M., Kato K., Saito Y., Yasuda K., Kagawa Y. (2004). Gene–nutrient and gene–gene interactions of controlled folate intake by Japanese women. Biochem. Biophys. Res. Commun..

[5] Zhang N., Zhou Z., Chi X., Fan F., Li S., Song Y., Zhang Y., Qin X., Sun N., Wang X. (2024). Folic acid supplementation for stroke prevention: A systematic review and meta-analysis of 21 randomized clinical trials worldwide. Clin. Nutr..

[6] Kagawa Y., Hiraoka M., Kageyama M., Kontai Y., Yurimoto M., Nishijima C., Sakamoto K. (2017). Medical cost savings in Sakado City and worldwide achieved by preventing disease by folic acid fortification. Congenit. Anom..

[7] Wang Z., Wang Z., Xu Y., Jiang J., Tang Y. (2022). B vitamins and prevention of cognitive decline and incident dementia: A systematic review and meta-analysis. Nutr. Rev..

[8] National Health Insurance Association National Health Insurance Database System 2015–2024.

[9] Ministry of Health, Labor and Welfare (2023). The National Health and Nutrition Survey Japan 2023.

[10] Saitama Prefectural Health Department (2022). Saitama Prefecture Specific Health Checkup Analysis Report 2022.

[11] Sakado City (2024). The 3rd Sakado City Healthy Town Development Plan.

[12] Yatsuda M., Furou M., Kamachi K., Sakamoto K., Shoji K., Ishihara O., Kagawa Y. (2025). Serotonin transporter gene polymorphisms predict adherence to weight loss programs independently of obesity-related genes. Nutrients.

[13] Nita R., Kawabata T., Kagawa Y., Shoji K., Nakayama K., Iwamoto S., Yanagisawa Y., Kimura F., Miyazawa T., Tatsuta N. (2025). Association of birth outcomes with maternal and infant FADS1 rs174547 genotypes in Japanese participants. Prostaglandins Leukot. Essent. Fatty Acids.

[14] Obata K., Segawa O., Yakabe M., Ishida Y., Kuroita T., Ikeda K., Kawakami B., Kawamura Y., Yohda M., Matsunaga T. (2001). Development of a novel method for operating magnetic particles, Magtration Technology, and its use for automating nucleic acid purification. J. Biosci. Bioeng..

[15] Kagawa Y., Hiraoka M., Miyashita-Hatano Y., Shishido-Oki M., Yoshida M., Kondou S., Sugiura M., Sawakami-Kobayashi K., Takahashi M., Tajima H. (2010). Automated single nucleotide polymorphism typing using bead array in capillary tube. J. Biosci. Bioeng..

[16] Han X., Pittman P., Ku L. (2021). The effect of National Health Service Corps clinician staffing on medical and behavioral health care costs in community health centers. Med. Care.

[17] Kagawa Y., Kagawa A., Lovenberg W., Yamori Y. (1984). Secondary prevention of cardiovascular diseases of outpatients of the nutrition clinic. Nutritional Prevention of Cardiovascular Diseases.

[18] Yang Y., Hozawa A., Kogure M., Narita A., Hirata T., Nakamura T., Tsuchiya N., Nakaya N., Ninomiya T., Okuda N. (2020). Dietary inflammatory index positively associated with high-sensitivity C-reactive protein level in Japanese from NIPPON DATA 2010. J. Epidemiol..

[19] Takano M., Hayashi F., Takemi Y. (2025). Associations between adherence to the Japanese Food Guide Spinning Top and nutrient density, climate impacts, and monetary cost: Findings from the Saitama Prefecture Nutrition Survey 2017. Eur. J. Nutr..

[20] Tabacchi G., Filippi A.R., Amodio E., Jemni M., Bianco A., Firenze A., Mammina C. (2016). A meta-analysis of the validity of FFQ targeted to adolescents. Public Health Nutr..

[21] Kobayashi S., Honda S., Murakami K., Sasaki S., Okubo H., Hirota N., Notsu A., Fukui M., Date C. (2012). Both comprehensive and brief self-administered diet history questionnaires satisfactorily rank nutrient intakes in Japanese adults. J. Epidemiol..

[22] Kagawa Y., Dever G.J., Otto C.T., Charupoonphol P., Supannatas S., Yanagisawa Y., Sakuma M., Hasegawa K. (2003). Single nucleotide polymorphism and lifestyle-related diseases in the Asia-Pacific region: Comparative study in Okinawa, Palau and Thailand. Asia Pac. J. Public Health.

[23] Hiraoka M., Kagawa Y. (2017). Genetic polymorphisms and folate status. Congenit. Anom..

[24] Saitama Prefectural Health Department (2011). The National Health and Nutrition Survey on Saitama Prefecture Residents 2011.

[25] Nakayama K., Bayasgalan T., Tazoe F., Yanagisawa Y., Gotoh T., Yamanaka K., Ogawa A., Munkhtulga L., Chimedregze U., Kagawa Y. (2010). A single nucleotide polymorphism in the *FADS1/FADS2* gene is associated with plasma lipid profiles in two genetically similar Asian ethnic groups with distinctive differences in lifestyle. Hum. Genet..

[26] Saitama Prefecture National Health Insurance (2025). Medical Expenses in Sakado City Compared with Those in Saitama Prefecture.

[27] Eto K., Nakanishi A., Fujikura J., Matsushita K., Tanaka H., Kagawa A., Takemi Y. (2019). Achievements and challenges of a Sakado “Shokuiku” program implemented in all elementary and middle schools in Sakado City, Saitama Prefecture. Nihon Koshu Eisei Zasshi.

[28] Dary O. (2006). Nutritional interpretation of folic acid interventions. Nutr. Rev..

[29] Huang T., Wahlqvist M.L., Li D. (2012). Effect of n-3 polyunsaturated fatty acid on gene expression of the critical enzymes involved in homocysteine metabolism. Nutr. J..

[30] Kume A., Kurotani K., Sato M., Ejima Y., Pham N.M., Nanri A., Kuwahara K., Mizoue T. (2013). Polyunsaturated fatty acids in serum and homocysteine concentrations in Japanese men and women: A cross-sectional study. Nutr. Metab..

[31] Bentley T.G., Weinstein M.C., Willett W.C., Kuntz K.M. (2009). A cost-effectiveness analysis of folic acid fortification policy in the United States. Public Health Nutr..

[32] Pujia C., Ferro Y., Mazza E., Maurotti S., Montalcini T., Pujia A. (2025). The role of mobile apps in obesity management: Systematic review and meta-analysis. J. Med. Internet Res..

[33] National Center for Health Statistics (NCHS) (2020). What We Eat in America, NHANES 2017–2018.

[34] Galekop M.M.J., Calder P.C., Baker E.J., Góralska J., Raźny U., Malczewska-Malec M., Uyl-de Groot C.A., Redekop W.K. (2025). Cost-effectiveness of personalized nutrition in adults with overweight and obesity: PREVENTOMICS Studies in Poland and the UK. J. Hum. Nutr. Diet..

[35] Pedroso I., Saravanan S.K., Kumbhare S.V., Sharma G., Almonacid D.E., Sinha R. (2025). Economic impact of a precision nutrition digital therapeutic on employer health costs: A multi-employer and multi-year claims analysis. Healthcare.

